# A Dissertation on the Endermic Method

**Published:** 1838-04

**Authors:** 


### Art. II.

*Dissertatio de Methodo Endermatico.* Auctore A. Ahrensen, m.d. —*Hauniæ*, 1836.

*A Dissertation on the Endermic Method.* By Dr. Ahrensen. —*Copenhagen*, 1836. 12mo. pp. 267.

When we consider the multitude of systems and theories which have been alternately devised and repudiated, and the variety of modes by which skilful and sagacious men have endeavoured to counteract the influence of noxious agencies on the human system, we are forcibly impressed with the truth of the Hippocratic apothegm, "Experience is fallacious, and judgment difficult." And we are led to be cautious, in yielding assent to new doctrines, or adopting new methods in the healing art, until their intrinsic value shall have been determined, not merely by the author himself, but by the concurrent testimony of other competent judges. It seems to have been in this philosophical spirit that Dr. Ahrensen has made the endermic method the subject of diligent and persevering investigation; examining all things, but holding fast only what he has confirmed by experimental research.

The mode of introducing medicaments by the skin is of very ancient date. Thus, to go no further, we read in Celsus, that a person in good health, "neque medico, neque iatrolipta egere,"* wants neither a physician nor a rubbing-doctor. The *iatraliptic method*, as we infer from its derivation, consisted in applying medicinal substances to the sound skin; some medicines being found to produce more powerful effects in that way than when administered internally; and these not only of a vegetable nature and easily soluble, but even metallic and almost insoluble matters. Thus, Haller states that Berengarius was the first person to discover that mercury was capable of exerting its specific effects through that channel; and that Amatus Lusitanus had observed grave symptoms consequent upon the external use of arsenic. Hippocrates and Galen describe the aperient effects of baths impregnated with hellebore; and Avicenna extols the efficacy of a bath of oil in resolving spasm.

By reflecting on the multiplied authenticated instances of the therapeutic effects so produced, the idea suggested itself to MM. Lembert and Lesieur, two Parisian physicians, that these would be enhanced provided the epidermic layers were previously removed, and the medicinal agent brought into direct contact with the cutaneous absorbing apparatus. This they denominated the *endermic method*, and the results of their original researches are recorded in a memoir which they published on the subject, in the year 1824. To an abstract of these Dr. Ahrensen has appended the subsequent observations of himself and others in the same field, so classed, grouped, and methodized as to form a comprehensive view of the whole.

After premising some observations on the structure and functions of the skin, in accordance with the recent discoveries of Breschet, Gurlt, and Wendt, the author proceeds:

* Lib. i. cap. i.

"Whence we see that the skin resembles, in many respects, 'the internal mucous lining of the alimentary canal. Each membrane consists of the same cellular tissue; and each is connected, although principally the latter, with organs containing numerous vascular and nervous ramifications. Both abound in organs of secretion, differing among themselves, and adapted to the leading functions of each; whose use in the economy does not, indeed, differ so much as we should at first view imagine, inasmuch as they both serve to depurate the organism, by relieving it of effete or obnoxious matters. Both are, moreover, furnished with a protecting envelope, and, if it be thicker in the skin, we nevertheless find even that typified in the compact epithelium constituting the inner surface of the stomach of granivorous birds. The analogy becomes still more palpable when we take into account the circumstance that mucous membrane, exposed for a length of time to the influence of the atmosphere, acquires the appearance and texture of skin; and that hairs have been detected growing from the mucous coat of the intestine." (P. 14.)

The author notices the arguments in proof of cutaneous absorption, based on the conclusive experiments of Bradner Stuart and Westrumb; and refutes the opinion of those writers who pretend that the therapeutic effects ascribed to cutaneous absorption ought to be exclusively referred to nervous agency or pulmonary inhalation. The transmission of substances through the skin is accomplished by the capillaries alone, by simple imbibition, or by imbibition conjoined with endosmosis; and rarely, unless some morbid irritation coexist, as Westrumb has endeavoured to shew, are substances at once taken up by the lymphatics, and conveyed into the current of the circulation. In the digestive canal, the ingesta fitted for assimilation, after undergoing certain changes, are received by the lymphatics, and whatever part withstands this necessary decomposition enters the blood in the way above pointed out, but more readily and promptly, inasmuch as the epithelium or mucous lining is more delicate than the epidermis, and capillaries are there present in greater number. According to Müller, saline solutions applied to the corium penetrate the capillaries in one second of time.*

The first step in the endermic method is the removal of the cuticle. This may be effected either with a common blister, covered over with a poultice to allay pain; with caustic ammonia, united with axunge; by means of a steel blade, heated by immersion in boiling water; or by the combustion of alcohol or ether, on a portion of lint of the requisite size. The selection of the spot for application will depend, in a great measure, on the nature of the remedy and the seat of the disease. Hence, morphia ought generally to be applied to the head; strychnia, near the vertebral column; belladonna, to the eye; emetics and laxatives, to the abdomen; diuretics, to the region of the kidney.

The medicines best suited for endermic use are such as exist in a concentrated form, as the alkaloïds, and are of a soluble and unirritating nature. They should be sprinkled on as a dry, impalpable powder. If the dose be very minute,—that is to say, considerably under a grain,—some gum or starch may be triturated along with it, in order to secure uniform diffusion. Remedies endowed with irritant qualities, as squill, kermes mineral, sulphate and muriate of quina, ought to be mingled with a little axunge or gelatine. With the exception of morphia and strychnia, the quantity employed ought considerably to exceed that

* Handbuch der Physiologie. Coblenz, 1834. Tome i. p. 233.

prescribed internally. The manner in which medicaments are to be brought into contact with the corium varies according to circumstances. In the first place, the epidermic vesicle is to be detached with a forceps, and the contained serum gently wiped away with a bit of soft sponge or lint. Nor is the lint to be allowed to remain on the raw surface for a certain time, as some propose, with the view of absorbing any fresh-formed serum, since its presence invariably creates irritation. Should there be any coagulable lymph deposited, it must, in like manner, be cleared off. These preliminaries over, the application may be made, unless the excoriation be inflamed, or speckled with small ecchymosed points, threatening gangrene.

"Powders, little irritant, are to be lightly dusted on the denuded corium, from their paper envelope. When applied on a sloping part of the body, they ought previously to be formed into a paste with water; a measure which aids absorption, as I ascertained by experiment: for, having selected some cases on purpose, I applied the muriate of morphia in one set, in the form of dry powder; in the other, moistened with water. On removing the bandages, after the lapse of twelve hours, I found, in the former, the fourth part of the mass still remaining unchanged; while in the latter it was wholly absorbed. The application made, the part is to be covered with a piece of lint, thinly spread with cerate, retained in apposition by means of strips of adhesive plaster and a bandage." (P. 62.)

The author remarks, that some writers on this subject recommend a simpler sort of dressing. It consists in covering the excoriated surface with a portion of wet blotting paper; which, on drying, adheres so firmly as to supersede the necessity of plasters or bandage. But, as its separation is apt to occasion pain and hemorrhage, and thereby prove detrimental to the subsequent application, it should in no case be resorted to without interposing a piece of waxed paper. The intervention of gauze has been found advantageous in employing acrid substances, as squill. An appropriate apparatus has been devised by M. Lesieur for remedies in the gaseous form. It is a vessel furnished with two opposite tubes fitted with stopcocks; the one for the admission, the other for the egress of the gas. On renewing the application, the medical attendant must first carefully remove any pseudo membrane formed on the denuded surface from the coagulated serum, which is often so thin and transparent as to be hardly visible to the naked eye.

In the endermic use of medicines, we must distinguish the local and primary from the general and secondary effects, in order to obtain a true estimate of their curative powers in any given disease. The local phenomena supervene promptly, and vary according to the physical properties and amount of the remedy. Frequently they consist of itching, with a slight sense of heat and tingling; sometimes, however, of violent agonizing pain; together with redness, more or less diffused, owing to the injection of the small vessels with blood. Various other symptoms have been enumerated by Lembert and Richter, none of which our author ever witnessed.

"The patients, they say, are seized with a sense of burning, darting pain or heat, which the last-named author affirms, if in the first instance the remedy applied be narcotic, extends itself in a radiating manner from the denuded point along the tract of the principal nerves in the vicinity, producing an universal elevation in the temperature of the body. Lembert, on the other hand, states that the painful sensations are first of all transmitted to the adjunct visceral cavities, and then, pursuing the tract of the vessels and nerves, spread over the body at large." (P. 69.)

The above-described effects form, as it were, the transition to the general effects, and last from ten or fifteen minutes to two or three hours.

The action of endermic remedies cannot be explained on the principle of sympathy; since it has been found that kermes mineral, sprinkled over a raw surface behind the ear, provokes expectoration; and belladonna, applied in the same manner to the foot, dilates the pupil. Neither can we shew, on the ground of sympathy, why affections of organs very different may be induced by different remedies applied to the same point of the skin. That there are instances in which the nervous system is immediately influenced by endermic remedies, is abundantly demonstrated by the sudden sedative effects, local as well as general, exercised by certain narcotics, before the vascular system has had time to be affected. Hence the opinion entertained by Lembert that medicines must be absorbed, either in whole or in part, ere any result can ensue, falls to the ground on his own shewing that sulphate of quina can check the paroxysm of ague in the course of ten minutes; that is to say, long before any adequate absorption can have taken place. It must not be supposed that we deny the agency of absorption; we wish merely to point out that there is a twofold action with narcotics, as proved by physiology and exemplified in the endermic method. For, if a suitable dose of morphia, or any of its salts, be directly applied to the diseased part, in order to allay topical pain, its anodyne effect is almost instantaneously perceived; at all events, half an hour before general symptoms, as somnolence and vertigo, occur: whereas, with the same application to some more remote part, the assuaging of the local pain is synchronous with the development of general symptoms.

The influence of absorption is, indeed, intimately connected with this branch of therapeutics; and whatever tends to diminish or augment that function will reciprocally interfere with the efficacy of endermic remedies. Hoffmann* has furnished some useful practical information on the subject, which is deserving of notice in this place. He observes, that it proceeds with greatest rapidity in individuals of a sanguine temperament, who have a soft and delicate skin, and in women and children; during evening and night; and in a moist warm temperature. It is most favoured when the functions appear to be all in equilibrium: wherefore, under any predominant affection of an organ or system, as anormal activity of the circulation, excessive plasticity of the blood, (as in inflammation, suppuration, pregnancy,) or great heats, high electric tension of the atmosphere, it is interrupted, limited, or hindered. It is likewise greater while the stomach is empty than during the process of digestion. The preponderance of any secretion, as in diarrhœa, running piles, partial sweats, diminishes absorptive energy, according to the same authority; but both physiology and pathology are in direct contradiction to such an assumption. Magendie's experiments clearly proved that, if the mass of circulating fluids be lessened, absorption is augmented in a corresponding degree.

Soemmering and Magendie assert that local inflammation diminishes

* Hufeland's Journal, January, February, 1833.

the force of absorption. Our author, on the contrary, maintains that slight inflammation or irritation is hardly, if at all, prejudicial.

"For I am convinced that, by reiterated application to the same blistered surface, remedies act more powerfully; and surgeons are well aware of the fact that many chronic tumours not only decrease in volume, but that even the organs in which these had been contained become atrophied by inflammation artificially established." (P. 72.)

Guided by the light of physiology, we at once see that the endermic inspersion is best performed in the evening, or else in the morning, ere the intestines be loaded with ingesta. Indeed, so close a relation subsists between the digestive canal and the skin, that, the more the former is tasked, the feebler will the function of the latter be.

In order to realize the benefit of a moist and warm temperature, Lembert conjoins the use of the tepid bath before the inspersion be made: "perhaps, says Dr. Ahrensen, a large poultice laid upon the denuded spot may in like manner prove advantageous, though in a less degree." He expresses surprise that compression and electricity, two agents of acknowledged utility in promoting absorption, should not as yet have been made subservient to the endermic method.

In reference to toxicology, we learn from the method in question, that morphia is the best antidote for strychnia, that musk and camphor counteract the narcotism of morphia; and that prompt ablution and detersion of the part, together with the application of the cupping-glass, will in most cases suspend and avert any baneful consequences.

The endermic plan of treatment is necessarily very circumscribed, inasmuch as it only admits of medicines of which a minimum dose will produce a powerful effect, and which are susceptible of ready absorption, or of exercising a decided effect on the superficial nerves. Hence the narrow range of materia medica it involves can derive extension alone from the future progress of chemical discovery. With the exception of narcotics and antispasmodics, whose curative virtues endermically administered are fully attested, but slender reliance can be placed on any other medicines.

"On this ground it appears that nervous are almost the only maladies which are completely under the control of the endermic method, and that only in as far as they are of dynamic origin and independent of organic disease: hence, the more local the affection, and the more superficial the portion of the nervous system implicated, the more rapid and efficient will be the result of the treatment. For this reason it is, that patients labouring under local neuralgia, paralysis, spasms, and periodical complaints, will be most speedily and certainly relieved." (P. 93.)

It may be granted, however, that some other remedies which act upon particular systems, as diuretics for example, will occasionally work a cure when endermically employed. Where there is a difficulty or impossibility of introducing medicine in the ordinary way, as in trismus or in affections of the fauces or œsophagus, which preclude access to the stomach, and in irritable states of that viscus, the endermic method is indicated. Its main advantages, however, are thus portrayed by Dr. Ahrensen.

"1. Medicines may be applied directly to the affected part, and by the consequent suppuration will act more or less as derivatives. This is of importance, as regards patients suffering from neuralgic pain and local spasm, as these may be immediately alleviated by narcotics without the patients experiencing any bad effects from the latter.

5

"2. As the stomach and intestinal tube are exempted from the irritation which may follow medicinal ingesta, these, in their turn, escape decomposition from the secreted fluids they would encounter in the first passages. Thus are their effects undisguised; a point of importance in reference to the researches of the physician and the pharmacologist. Lesieur unphilosophically pretends that we shall be able to discriminate in this way the active constituents of remedies, by submitting the residuum to chemical analysis.

"3. The odour, taste, or perverse action of particular medicines offers no contra-indication here, since they can be exhibited without the knowledge of the patient. This is especially useful in the case of children, provided the medicine be not of too irritant a nature.

"4. This method enables us to extend the employment of dangerous remedies by administering much larger doses than we could venture upon internally, seeing that the means of obviating their deleterious effects are more within our reach.

"5. Where medicines given internally, from long continuance, cease to act, and where we cannot safely augment the dose, we may advantageously resort to the endermic plan, as a means of mitigating suffering in chronic and incurable diseases."* (P. 94-6.)

Among narcotics, the salts of morphia hold the first place. The acetate, sulphate, and muriate have been employed; but the two last are preferable from their superior solubility. Their endermic application is attended by an itching, or smarting, and sense of heat, which seldom lasts beyond a quarter of an hour. In sensitive females, however, it will sometimes continue for four or five hours. According to Richter, acetate of morphia does not produce either inflammation or increased secretion in the denuded skin; but Dr. Ahrensen, on the contrary, states that, if the salt be frequently applied, suppuration ensues, and a new epidermis is formed on the fourth or fifth day. The general effects resulting from their external, differ from those following their internal use, both as regards time and manner of acting. Lembert somewhat poetically compares their rapidity of operation to the swiftness of lightning; and Trousseau and Bonnet expressly state, that phenomena were manifested in two minutes' time after the endermic application, which took days according to the usual way, partly owing to the decomposing power of the stomach, partly to the prevailing absorption of the skin.

"Such prompt effects were seldom observed by Hoffmann, who reckons the interval of time to range between ten minutes and six hours. Although I do not dispute the greater celerity of the endermic plan, I have in no case witnessed symptoms indicative of the agency of morphia earlier than five minutes, (and there the large dose of a grain or a grain and half had been employed;) and frequently have I known two hours elapse ere the general symptoms made their appearance. The variation in time depends not only on the sensibility of the patient and the dose of the narcotic, but, as above mentioned, on the nature of the disease and the site of the application. If the organism be saturated, so to speak, with morphia from protracted use, a minute quantity then administered will act with greater energy and speed." (P. 102-3.)

Some writers contend that the excellence of the endermic application of morphia consists in its affording relief without the brain being consentaneously involved; but it is seldom that the brain remains free, and that only where a small dose, not exceeding the fourth or the half of a grain, has been applied directly to a part remote from the head. Otherwise,

* A case in point is related by Hoffmann, who, by alternately administering anodynes by the mouth and skin, succeeded in a remarkable manner, in relieving the tortures of a woman labouring under carcinoma.

we have, in addition to the narcotic symptoms, signs of cerebral disturbance. These may be of a mild form, as cephalalgia, vertigo, slight stupor, with pupils dilated or contracted, and nausea; or they may assume a more serious character, as great stupor, bilious vomiting, lively delirium, and tremors of the limbs remaining for days after; succeeded by more or less irritation of the vascular system, which, however, Lembert and Hoffmann deny; and, lastly, the secretion and excretions become altered in different ways. Morphia, as is well known, is very apt, when given internally, to nauseate the stomach; and Dr. Ahrensen ascertained that, if the endermic dose exceeded three quarters of a grain, squeamishness was induced; and, with a grain and a half, vomiting, constipation, ischuria, eruption of sweat, pruritus of the skin about the nose, and an occasional papular efflorescence.

The dose of morphia ought, in the first instance, to be small, if used near or on the head, not exceeding one-fourth or half a grain in adults; for children, one-sixteenth of a grain will suffice. If not found to disagree with the patient, it may be gradually increased to two, three, or even five grains. It has been found useful in the following diseases: Traumatic tetanus (conjoined with antiphlogistic treatment, if there be signs of inflammation present;) delirium tremens; mania; hysteria; spasmodic dysphagia; spasmodic cough; spasm of the eyelids; neuralgia—frontal, temporal, alveolar, and intermittent; pleurodyne; venereal pains; cancer; chronic rheumatism, when unattended with fever, of the kind termed fibrous, and of a local description, or in the instance of acute articular rheumatism confined to a single joint; chronic bronchitis; vomiting and cardialgia; dysuria; ague.

The remedy next in order is belladonna.

"It was first applied by Lembert; but, as it occasions, in the pure form, both irritation and violent pain, which Richter has known to last for an hour, it is always advisable to temper it with lard. It is said to affect the voice in a peculiar way, giving rise to a kind of involuntary stammering; and, if the quantity applied be large, and not far distant from the head, the pupil of the opposite side is usually dilated. Bally observed dilatation of the pupil, even when applied to the back of the foot. We find also delirium with loquacity, (the tongue, however, is slow,) with universal tremors; bilious vomiting, colic pains, diarrhœa, and abdominal distention, have been noticed by the same author." (P. 159.)

According to Gerrhard, belladonna, endermically used, is anodyne and antispasmodic, and diminishes the mucous secretion of the bronchi. Richter found a liniment, consisting of a scruple of the extract to two drachms of *aqua lauro-cerasi*, smeared over the denuded corium, answer well in irritable states of the air-passages, spasmodic vomiting, and gastrodynia. The extract of hyoscyamus has been endermically prescribed in a similar formula to the preceding. The extract of belladonna is generally employed, the root also sometimes. It is rich in atrophine.

Extract of stramonium occasions great pain. It is said to prove serviceable in obstinate neuralgia and sciatica; and Gerrhard has applied a scruple at a time to the perineum. Crocus, in a dose of six grains, is reported to have cured a case of intermittent occipital neuralgia.

Strychnia in the pure state was first endermically applied by Lembert and Lesieur; in consequence, it is said, of the favorable result which followed the insertion of some extract of nux vomica into a wound, in a case of palpebral paralysis under the care of M. Marjolin. The salts of strychnia, being more soluble, are for that reason preferable to the alkaloid itself, in an endermic point of view.

"If we look to the local effects of strychnia, we shall find them much more energetic than those of morphia; the pain following the application is burning and pungent, lasting about half an hour or an hour. Where blisters have been applied to the paralyzed limbs or the vertebral column, the raw surface inflames under the use of the remedy, and affords a copious suppuration, so much so as to require a repetition of the blister on the sixth day. Whatever way the remedy be used, its general effects are equally violent, and always directed to the medulla spinalis and ganglionic system. Endermically administered, we can persevere longer in its use, and convey a greater quantity into the system, with less danger of poisoning the patient." (P. 165.)

After some days' continuance, strychnia frequently causes painful spasmodic contractions of the extremities, resembling tetanus, disorder in the first passages, and vomiting. According to Richter, when the dose amounted to three grains, the patients complained of obstinate constipation, anorexia, heat of stomach, and squeamishness. From an experiment made by our author, we are authorized in considering strychnia a cumulative medicine. Its prolonged use sometimes gives rise to a peculiar modification of the sensibility, so that the characteristic spasms are apt to recur some time after the patient has left it off, on his taking violent exercise, or doing any thing to quicken the circulation of the blood. The endermic dose of pure strychnia should not, at the commencement, exceed half a grain, and of its salts one-fourth of a grain. It has been used with advantage in partial paralysis, especially in the paralysis from lead, and wherever we have no reason for suspecting the existence of organic disease. In hemiplegia, it has occasionally proved serviceable; as also in amaurosis, depending on paralysis of the optic nerve or retina, and in symptomatic amaurosis from some lesion of the fifth nerve; in hysteric aphonia and stammering; in prosopalgia, and other neuralgic affections; in chorea sancti viti. It is a curious circumstance that universal tremors often supervene contemporaneously with the melioration of the disease under treatment.

Veratrine and aconitine, from the intolerable pain they determine, are inappropriate for endermic use; but their therapeutic virtues can be adequately obtained by the *iatroleptic* method: our readers are well aware how much they have been employed in this way, since the publication of Dr. Turnbull's work.

Musk is the antispasmodic standing first on the endermic list. It has been administered in doses of six, ten, or fifteen grains, with good effect, in asthma. Other substances in the same class, castor, assafœtida, oxide of zinc, have been likewise tried, but do not seem to have answered.

Among endermic febrifuges, quina, together with its sulphate and muriate, stand forth pre-eminent.* Of the local effects of the latter we are told that, "soon after the inspersion of the powder, a violent burning pain ensues, which annoys the patient from fifteen minutes to an hour; nay, sometimes several hours; the skin acquires a red blush round the

* Hufeland (*über einige Kinderkrankheiten*, Leipzig, 1792,) relates instances of the efficacy of cinchona frictions in ague; which is confirmed by Auber and Chrestien. The *iatroleptic* powers of the muriate of quina, in infantile intermittents, are instanced by Michaelis. (*Magnusson de Methodo Endermatica*, p. 13.)

denuded spot, which, on the following day, is covered with a soft, yellow crust, dotted with white points, and consisting of particles of the salt imbedded in coagulable lymph; frequently tedious ulcers are left, which do not heal before several weeks."

Richter tells us he has known the skin deeply eroded by the action of the salts of quina. The pain is obviated by mixing the powder with axunge or sulphate of morphia. Its general effects are to increase the heat of the surface of the body, and the muscular and nervous energy. The salt of quina ought to be applied to the epigastrium during the apyrexia in ague, in the quantity of four or six grains. The blister should not be put on during the paroxysm.

"From a careful survey of numerous cases detailed by me, it clearly appears, that not only simple intermittent fevers frequently yield under the endermic use of this remedy, and that with a less dose than if internally exhibited; but complicated cases are more manageable in the same way." (P. 225.)

It has been also employed with benefit in intermittent nervous ailments. Iodide of quina, piperinum, salicinum have been severally put to the test of experiment, but without affording any satisfactory results.

Expectorants constitute the next class of endermic remedies which claim our attention. Kermes mineral, sesqui-sulphide of antimony, applied to a blistered surface on the lower part of the neck, (*quere*, the suprasternal fossa?) or to the chest, serves as an expectorant, in the dose of a grain, in pulmonary catarrh, in chronic bronchitis, and, alternated with morphia, in phthisis. It is a powerful topical irritant, and causes severe pain; hence it should not be employed in children. Its acrid qualities may be abated, however, by combination with axunge. Powdered kermes, according to Lembert, tends to concrete into hard incrustations on the corium, and their removal is not unattended with pain. Dr. Ahrensen, on the other hand, has seen rather a copious suppuration in consequence.

No reliance can be placed on the endermic effects of emetina, or tartarized antimony; yet Lettsom witnessed diaphoresis and diarrhœa, and Brera* vomiting, produced by inunction of the latter medicine.

*Diuretics.* Squill has been endermically administered in the form of the powder of the bulb, and of extract. The dose of the former is ten or twenty grains, twice or thrice a day; of the latter one or ten grains. The remedial effects are manifested, whether the substance be applied to the abdomen, lumbar region, or the arm.

"The local effects of both preparations are extremely irritant, but chiefly the extract; for the pain resulting from the powder subsides in general in from sixty to ninety minutes, while that from the extract may abide eight or ten hours. There is diffused redness, followed by inflammation and suppuration, in the place of application, which prevents the repetition of the remedy for more than two or three days consecutively to the same blister. Others, I believe, have persevered longer. The general effects do not differ materially from those usually attributed to its internal exhibition; some writers are of opinion that irritation of the alimentary canal is thus avoided; others, again, deny this; as M. Huss, who has known nausea and urgent vomiting incidental to its endermic application. Its diuretic virtues are established by the endermic method, both in my own experience and by that of others. It also promotes expectoration and transpiration." (Pp. 242-3.)

Its employment is indicated in anasarca and hydropic collections;

* Anatripsologie v. Brera, Wien 1800, p. 170.

but, owing to the vehemence of its local effects, it ought never to be prescribed, except the first passages be disordered.

Digitalis may be endermically prescribed, in the pulverized form, to the extent of eight or ten grains in the day. The denudation of the corium ought to be as near as possible to the affected organ.*

"The local effects of digitalis are sharp pains, which continue from fifteen minutes to half an hour, but are rarely so troublesome as to require the suspension of the medicine. The excoriated surface begins, in the course of a few days, to discharge matter, and in a fortnight is quite dried up. Its protracted use determines, as Raciborski has observed, a green coloration of the derma, resembling nævus, which abides for weeks, unless repeated washing has been resorted to. Its general effects, in whatever way administered, are nearly identical,—namely, depression of the vascular system, augmentation in the secretion of urine, together with the absorption of extravasated serum: these are seldom manifested before the lapse of two or three days, and, as happens with its internal exhibition, persist after the cessation of the remedy. Its external administration seems to exert a greater influence on the functions of the skin, as evinced by the increased transpiration; and is unattended by vomiting and diarrhœa, or any derangement of the alimentary canal or brain, incidental to its internal exhibition. And these circumstances are not unimportant; as Andral uniformly found that, where digitalis disagreed with the stomach, the vascular system was little depressed." (P. 249-250.)

This remedy is chiefly indicated in those cases where we desire to make a powerful impression on the heart and vascular system; as in simple hypertrophy of that organ, or in hypertrophy associated with valvular disease; Bouillaud extols its employment in tertian ague.

According to Gerrhard and Coster, iodine endermically applied, exerts a diuretic action. This our author refuses to admit.

Concerning purgative medicines, we find, from the concurrent testimony of most authors on the endermic method, that jalap, elaterium, colocynth, croton oil, and gamboge, irritate more or less the part to which they are applied, but do not determine any cathartic action;† as to rhubarb, it is perfectly inert. The remaining endermic remedies of which Dr. Ahrensen treats are aloes and calomel. Aloes applied to the denuded skin causes burning heat, and subsequently promotes healthy suppuration, and is laxative. A dose of ten grains is followed by an evacuation from the bowels in the course of half an hour, and by six or eight more during the next eight hours. The watery extract of aloes, applied to the epigastric region in the quantity of two to six grains, has been found effectual in relieving obstinate constipation where other remedies have failed; and in icterus.

Calomel, on account of its desiccative qualities, requires to be previously mixed up with sugar or axunge. The sense of heat following its endermic application is very transient. In the dose of three or four grains, it produces, it is said, a copious alvine discharge. The antisyphilitic virtues of calomel thus administered for a length of time, in the quantity of one-sixteenth to two grains, are very questionable.

"Corrosive sublimate, which, from its caustic nature, appears ill adapted to the

* The *iatroleptic* use of squill and digitalis was first noticed by Auber and Brera. (*Op. cit.*)

† Yet, Van Swieten comments upon the perils from hypercatharsis, to which persons exposed themselves in his time, by applying plasters and ointments to the epigastric region in order to move the bowels, containing *colocynth, scammony, aloes, and jalap.*

endermic method, has been nevertheless found useful by Bally in syphilitic exostosis and osteocopic pains. Reynaud cures bubo by applying to the blistered surface a compress moistened with a solution, containing ten grains of sublimate to an ounce of distilled water." (P. 267.)

On taking a retrospect of the results heretofore obtained by the endermic treatment of disease, most sensible persons will, we think, agree with us, that the few advantages it may offer are hardly sufficient to countervail the uncertainty and inconvenience with which it is fraught. If we except, perhaps, morphia, strychnia, squill, and aloes, no other medicines remain of which we can predicate any positive therapeutic effect; and the annoyance or irritation concomitant even upon their application, in many instances, will militate against any general introduction of the practice.

It would be doing injustice to Dr. Ahrensen to close this analysis of his treatise without expressing our high opinion of its merits. He has shown much industry and judgment in selecting his materials, and in appreciating the positive and relative value of the remedies treated of. His book, like most of those that issue from the medical school of Copenhagen, bears the stamp of good sense and sound learning.

---

